# Age‐ and sex‐related dietary specialization facilitate seasonal resource partitioning in a migratory shorebird

**DOI:** 10.1002/ece3.7175

**Published:** 2021-01-20

**Authors:** Laurie A. Hall, Susan E. W. De La Cruz, Isa Woo, Tomohiro Kuwae, John Y. Takekawa

**Affiliations:** ^1^ San Francisco Bay Estuary Field Station Western Ecological Research Center U.S. Geological Survey Moffett Field CA USA; ^2^ Coastal and Estuarine Environment Research Group Port and Airport Research Institute Yokosuka Japan; ^3^ Present address: Suisun Resource Conservation District Suisun City CA USA

**Keywords:** biofilm, competition, dietary specialization, microphytobenthos, resource partitioning, shorebirds

## Abstract

1. Dietary specialization is common in animals and has important implications for individual fitness, inter‐ and intraspecific competition, and the adaptive potential of a species. Diet composition can be influenced by age‐ and sex‐related factors including an individual's morphology, social status, and acquired skills; however, specialization may only be necessary when competition is intensified by high population densities or increased energetic demands.
2. To better understand the role of age‐ and sex‐related dietary specialization in facilitating seasonal resource partitioning, we inferred the contribution of biofilm, microphytobenthos, and benthic invertebrates to the diets of western sandpipers (*Calidris mauri*) from different demographic groups during mid‐winter (January/February) and at the onset of the breeding migration (April) using stable isotope mixing models. Western sandpipers are sexually dimorphic with females having significantly greater body mass and bill length than males.
3. Diet composition differed between seasons and among demographic groups. In winter, prey consumption was similar among demographic groups, but, in spring, diet composition differed with bill length and body mass explaining 31% of the total variation in diet composition. Epifaunal invertebrates made up a greater proportion of the diet in males which had lesser mass and shorter bills than females. Consumption of Polychaeta increased with increasing bill length and was greatest in adult females. In contrast, consumption of microphytobenthos, thought to be an important food source for migrating sandpipers, increased with decreasing bill length and was greatest in juvenile males.
4. Our results provide the first evidence that age‐ and sex‐related dietary specialization in western sandpipers facilitate seasonal resource partitioning that could reduce competition during spring at the onset of the breeding migration.
5. Our study underscores the importance of examining resource partitioning throughout the annual cycle to inform fitness and demographic models and facilitate conservation efforts.

## Graphical abstract

Our results provide the first evidence of seasonal differences in diet composition among demographic groups of western sandpipers, supporting the conclusion that age‐ and sex‐related dietary specialization facilitate seasonal resource partitioning in this species. Differences in diet composition among demographic groups were more pronounced at the onset of migration in spring compared to mid‐winter following the expectation that the consumption of biofilm and microphytobenthos provides energy for sandpipers during their breeding migration and reduces competition when high densities of birds occupy foraging sites. In spring, juveniles, particularly juvenile males, consumed more biofilm and microphytobenthos than adults supporting our hypothesis that sandpipers with shorter bills, lesser mass, and less prey handling experience would consume the greatest amount of biofilm and microphytobenthos. Photo credit: William Chan.

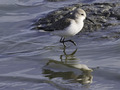

## 1 INTRODUCTION

Dietary specialization—the tendency of individuals to forage on specific prey items that differ from the prey of other individuals in their population—is common in animals and has important implications for individual fitness, inter‐ and intraspecific competition, and the adaptive potential of a species (Bolnick et al., 2003; Durell, 2000). Age and sex play a particularly important role in influencing dietary specialization, which has been well‐studied in the shorebirds and their allies (Charadriiformes; Bocher et al., 2014; Catry et al., 2012; Durell, 2000, 2003; Jehl & Murray, 1986; Székely et al., 2004).

Age‐related factors including morphology, social status, and acquired skills can influence dietary specialization in shorebirds (Durell, 2000, 2003). Juvenile birds tend to have smaller bodies and shorter bills than adults, and, in some shorebird species, juveniles consume smaller prey (Fasola et al., 1996; Goss‐Custard & Durell, 1987; Puttick, 1978). Juvenile birds may also be excluded from foraging in areas with the most profitable prey because of their subdominant social status (Durell, 2003). In addition, dietary differences between juveniles and adults could arise because juveniles lack the skill needed to acquire and handle certain prey (Durell, 2000, 2003).

Morphological differences between sexes can also influence dietary specialization in shorebirds. Many shorebirds exhibit sexual dimorphism in body size and bill length (Jehl & Murray, 1986; Székely et al., 2000, 2004). Body size dimorphism is thought to be driven by sexual selection (Jehl & Murray, 1986; Székely et al., 2000, 2004). It may also have energetic consequences that impact prey selection because larger‐bodied birds require more calories to sustain their greater basal metabolic rates (Lindström & Klaassen, 2003). Further, larger‐bodied birds could exert dominance over smaller‐bodied birds and monopolize profitable prey or foraging areas (Alves et al., 2013; Both et al., 2003). Sexual dimorphism in bill length is more pronounced than in other body parts and is thought to be a consequence of competition for food during the nonbreeding season (Jehl & Murray, 1986; Székely et al., 2000, 2004). Several studies have demonstrated that bill size dimorphism in shorebirds results in resource partitioning between sexes (Alves et al., 2013; Catry et al., 2012; Recher, 1966; Stein et al., 2008).

The western sandpiper (*Calidris mauri*) is a small (22–35 g) migratory shorebird that exhibits female‐biased sexual size dimorphism with a 5% greater body size and a 15% longer bill in females compared to males (Jehl & Murray, 1986; Page & Fearis, 1971). Along the Pacific coast of North and South America, the nonbreeding range of the species extends from British Columbia, Canada to Mollendo, Peru (Franks et al., 2020). The western sandpiper is considered a generalist with a diverse diet that includes benthic invertebrates and biofilm—a thin layer of microphytobenthos, bacteria, and detritus encased in a polysaccharide‐rich matrix of extracellular polymeric substances that forms on the surface of mudflats (Stal, 2003; Underwood & Paterson, 2003). Biofilm is an important food for migrating western sandpipers during spring, but little is known about biofilm consumption during mid‐winter or whether biofilm consumption differs among sexes and age classes (Kuwae et al., 2012; Schnurr et al., 2019, 2020).

During the nonbreeding season, dietary specialization among western sandpipers would allow sandpipers to partition resources and reduce inter‐ and intraspecific competition for prey; however, specialization may only be necessary when competitive pressure is intensified by high population densities or increased energetic demands, such as during the breeding migration in spring (Svanbäck & Bolnick, 2005). To better understand the role of age‐ and sex‐related dietary specialization in facilitating seasonal resource partitioning, we quantified the contribution of biofilm, microphytobenthos, and benthic invertebrates to the diets of western sandpipers from different demographic groups during mid‐winter (January/February) and at the onset of the breeding migration (April). Because smaller‐bodied, shorter‐billed male western sandpipers spend a significantly greater proportion of time pecking to consume benthic epifauna at the surface compared to females, we hypothesized that males would consume more biofilm and microphytobenthos than females (Fernández & Lank, 2008; Mathot & Elner, 2004; Mathot et al., 2007; Nebel, 2005; Nebel et al., 2002). We also hypothesized that juveniles would consume more biofilm and microphytobenthos than adults because juveniles are expected to have less prey handling experience than adults (Durell, 2000, 2003). In addition, we hypothesized that biofilm and microphytobenthos consumption would be greater in spring at the onset of migration than during mid‐winter based on the expectation that sandpipers consume biofilm and microphytobenthos to help fuel their migration and reduce competition during periods when high densities of birds occupy foraging sites (Kuwae et al., 2012; Schnurr et al., 2019, 2020).

## 2 MATERIALS AND METHODS

### 2.1 Study area

The San Francisco Bay (SF Bay) is the largest estuary on the west coast of North America. It is designated as a Western Hemisphere Shorebird Reserve Network site of hemispheric importance because it is visited by over half a million wintering and migratory shorebirds each year (Morrison, 1981; Myers et al., 1987; Page et al., 1999). Western sandpipers are one of the most abundant shorebird species in SF Bay, foraging on tidal mudflats throughout the nonbreeding season (Page et al., 1999). Western sandpipers exhibit strong foraging site fidelity and have similar home range sizes in winter and spring in this region (Warnock & Takekawa, 1996). In addition to serving as an important wintering area for western sandpipers, SF Bay is a heavily utilized migratory stopover site for sandpipers wintering at more southern latitudes (Bishop et al., 2006; Butler et al., 1996; Iverson et al., 1996). The influx of migratory western sandpipers in April leads to increased population densities and rapid depletion of invertebrate prey that could increase competition (Rowan, 2012). Our study was conducted on the Dumbarton shoal, an intertidal mudflat on the southwestern side of SF Bay. This mudflat supports a high biomass of benthic invertebrates and biofilm consumed by western sandpipers, but the invertebrate carrying capacity is exceeded in April (Rowan, 2012).

### 2.2 Sample collection and preparation

Western sandpipers (*n* = 97) were captured with mist nets during mid‐winter (23 January, 22 February, and 24 February) and at the onset of the breeding migration in spring (19–23 April) of 2012 (Table S1). Each bird was aged (after‐hatch‐year = adult, second‐year = juvenile) and sexed based on plumage characteristics and morphometric measurements so that it could be assigned to one of four demographic groups (adult female, adult male, juvenile female, juvenile male; Page & Fearis, 1971; Pyle, 2008). Approximately 250 µl of blood was collected from the brachial vein in the wing and transferred to a heparinized microcentrifuge tube. Blood was centrifuged in the field for 5 min at 5,000 rpm to separate fractions of plasma and red blood cells, which were transported on ice and stored at −20°C until they were freeze‐dried.

Biofilm (*n* = 18), microphytobenthos (*n* = 18), and benthic invertebrates (*n* = 83) were collected on the mudflat in January and April of 2012 (Table S1). We included biofilm and microphytobenthos as separate prey groups because evidence suggests that sandpipers are able to selectively feed on the microphytobenthic component of biofilm, though they inevitably ingest some whole biofilm (Kuwae et al., 2008). Biofilm and microphytobenthos samples were collected at nine locations along a 450‐m transect that was perpendicular to shore. At each location, one biofilm and one microphytobenthos sample were collected from the sediment surface by scraping the top 2 mm from 10‐cm diameter circular plots into plastic bags. Invertebrates visible to the naked eye were removed from the samples. Samples were transported on ice. Microphytobenthos samples were refrigerated (4°C) overnight, and the microphytobenthos was extracted the next day following the protocol of Kuwae et al. (2008, 2012). Biofilm was stored at −20°C and was later thawed and dried for 3–4 days in a dark room. A 4‐g sample of biofilm and microphytobenthos were each sent for stable isotope analysis. To collect benthic invertebrates, surface sediments (≤5 cm deep) were collected into plastic bags, transported on ice, and refrigerated (4°C). Within two days, sediments were rinsed through a 500 µm sieve. Five classes of invertebrates (Bivalvia, Clitellata, Gastropoda, Malcostraca, and Polychaeta) were retained for stable isotope analysis based on their availability in our study area and previous studies of western sandpiper diet (Mathot et al., 2007; Sutherland et al., 2000). Invertebrates were dried to constant mass, and individuals from each class were pooled to achieve a sample mass of approximately 4 g.

### 2.3 Stable isotope analysis

Analyses of *δ* ^13^C and *δ* ^15^N values in western sandpiper plasma, biofilm, microphytobenthos, and invertebrates were conducted at the Port and Airport Research Institute (Nagase, Yokosuka, Japan). Plasma samples were homogenized with a microspatula, and biofilm, microphytobenthos, and invertebrate samples were homogenized with a mortar and pestle. Samples were weighed and packed into tin capsules. Isotope ratios were measured with a Thermo Electron Delta Plus Advantage gas isotope ratio mass spectrometer (Bremen, Germany) interfaced with a Thermo Electron FlashEA 1112 elemental analyzer (Bremen, Germany). The long‐term analytical precision was <2‰ for *δ* ^13^C and *δ* ^15^N. Vienna Pee Dee Belemnite (VPDB) and air (AIR) were used as standards for *δ* ^13^C and *δ* ^15^N, respectively. l‐Histidine and l‐Alanine (Shoko Science Co., Ltd.) standards and blanks were included with each run to correct for drift. Isotope ratios are reported in parts per thousand (‰) using delta notation with *δ* ^h^ *N* = (*R*_sample_/*R*_standard_ − 1) × 1,000, where *R* is the ratio of enriched to depleted isotopes for the sample or standard, *N* is the element of interest, and *h* is the mass of the enriched isotope. Most samples had C:N ratios > 4. Therefore, we normalized *δ* ^13^C values to account for variable lipid content according to the following equation from Post et al. (2007):

$$\delta^{13}C_{\mathrm{normalized}}=\delta^{13}C_{\mathrm{original}}‐3.32+0.99\times C:N$$

Normalized *δ* ^13^C values were used in all subsequent analyses.

Plasma was selected for stable isotope analysis because of its rapid turnover rate. Turnover rates for carbon and nitrogen isotopes in western sandpiper plasma are unknown; however, *δ* ^13^C and *δ* ^15^N values reached equilibrium in dunlin (*Calidris alpina*) plasma after 10 days following a diet switch. The dunlin is a close relative of the western sandpiper, and it is likely that isotopic turnover occurs more quickly in western sandpiper plasma because western sandpipers have 50% less body mass and faster metabolic rates than dunlin (Lourenço et al., 2015). Western sandpipers captured in SF Bay in April could have wintered in SF Bay or migrated to SF Bay from wintering areas to the south. Migrants typically arrive in mid‐March and early‐April with an average duration of stay in SF Bay of 12 days (Iverson et al., 1996). The average departure date from SF Bay for western sandpipers is 28 April (range = 20 April–9 May; Bishop et al., 2006). Our captures from 19 to 23 April coincided with the onset of western sandpiper departures from SF Bay, at which time most birds would have plasma at isotopic equilibrium with SF Bay prey.

### 2.4 Statistical analysis

All statistical analyses were performed in R v. 3.6.1 (R Core Team, 2019). For each season, we estimated differences in *δ* ^13^C and *δ* ^15^N values of western sandpiper plasma among demographic groups using general linear models (GLMs) with the *lm* function. To characterize the diet of western sandpipers in each season, we first estimated differences in *δ* ^13^C and *δ* ^15^N values among five groups of prey: biofilm, microphytobenthos, Bivalvia, Polychaeta, and other invertebrates (Clitellata, Gastropoda, and Malacostraca) using a linear discriminant analysis (LDA). Clitellata, Gastropoda, and Malacostraca were grouped into a single prey source because there was substantial overlap in their *δ* ^13^C and *δ* ^15^N values. Statistical significance of *δ* ^13^C and *δ* ^15^N values in the LDA was determined with forward stepwise selection using Wilks’ Lambda (λ) criterion in the klaR package (Weihs et al., 2005). We then estimated the proportional contribution of the five prey groups to the diets of western sandpipers using a stable isotope mixing model in the IsotopeR package (Hopkins & Ferguson, 2012). Model runs were conducted with three chains, a burn‐in of 100,000 iterations, a run of 100,000 iterations, and a thinning rate of 100. We compared the results of two mixing models using discrimination factors for plasma derived from dunlin from two previously published studies: Δ^13^C ± standard deviation (*SD*) = 0.50 ± 0.42‰, Δ^15^N ± *SD* = 3.30 ± 0.32‰ (Evans Ogden et al., 2004) and Δ^13^C ± *SD* = 0.32 ± 0.16‰, Δ^15^N ± *SD* = 3.30 ± 0.20‰ (Lourenço et al., 2015). Discrimination factors were added to the stable isotope values of all prey prior to model runs. Discrimination factors have been shown to vary among different environments, trophic levels, taxa, tissues, metabolic rates, modes of nitrogenous excretion, sample preparations, and food sources with different qualities and quantities of protein (Dalerum & Angerbjörn, 2005; Florin et al., 2011; McCutchan et al., 2003; Robbins et al., 2010; Vanderklift & Ponsard, 2003). To help account for this variation, we incorporated uncertainty in discrimination factors by including standard deviation in our mixing models (Hopkins & Ferguson, 2012).

We compared the diet composition of western sandpipers obtained from the mixing models between seasons, among demographic groups, and among demographic groups within each season using a Permutational Analysis of Variance (PERMANOVA) with 9,999 permutations based on Bray–Curtis dissimilarities in the Vegan package (Oksanen et al., 2019). We ensured homogeneity of multivariate dispersion using permutest in Vegan (Oksanen et al., 2019).

Finally, we examined the relationship between morphological traits of individual western sandpipers and diet composition using redundancy analysis (RDA). We estimated the proportion of variance in diet composition explained by body mass and bill (culmen) length. The RDA was conducted using forward selection with 10,000 permutations using ordistep in Vegan (Oksanen et al., 2019). Differences in body mass and bill length among demographic groups were assessed using GLMs.

## 3 RESULTS

**FIGURE 1:**
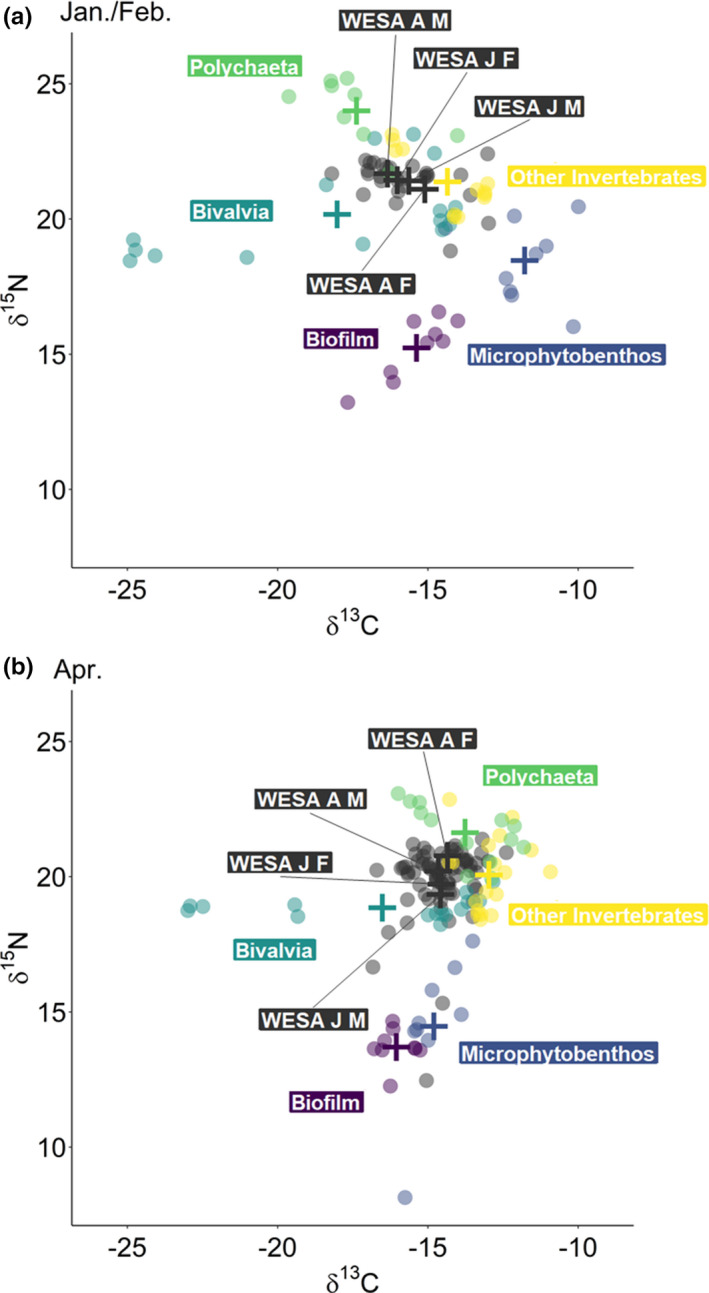
Stable *δ* ^13^C and *δ* ^15^N isotope values of western sandpiper (*Calidris mauri*; WESA A F = adult female, WESA A M = adult male, WESA J F = juvenile female, WESA J M = juvenile male) plasma and five prey groups collected in (a) January/February and (b) April in San Francisco Bay, CA, USA. Prey isotope values were adjusted by adding discrimination factors for dunlin (*C. alpina*) plasma from Evans Ogden et al. (*Δ* ^13^C ± standard deviation (*SD*) = 0.50 ± 0.42‰, *Δ* ^15^N ± *SD* = 3.30 ± 0.32‰; 2004). Means for each group are displayed as crosses. See Table S1 for sample sizes

Western sandpiper plasma did not differ significantly among demographic groups in *δ* ^13^C (all *p* > .05) or *δ* ^15^N (all *p* > .05) in mid‐winter (Figure 1). In spring, *δ* ^13^C (all *p* > .05) values did not differ among demographic groups, but *δ* ^15^N values were significantly lesser in juvenile males compared to adult females (*p* = .04; Figure 1). In each season, stable isotope signatures differed among the five prey groups (Figure 1; Figure S1). Only *δ* ^15^N contributed significantly to the linear discriminant function that differentiated prey groups in winter (*λ* = 0.18, *p* < .001) and spring (*λ* = 0.17, *p* < .001).

**FIGURE 2:**
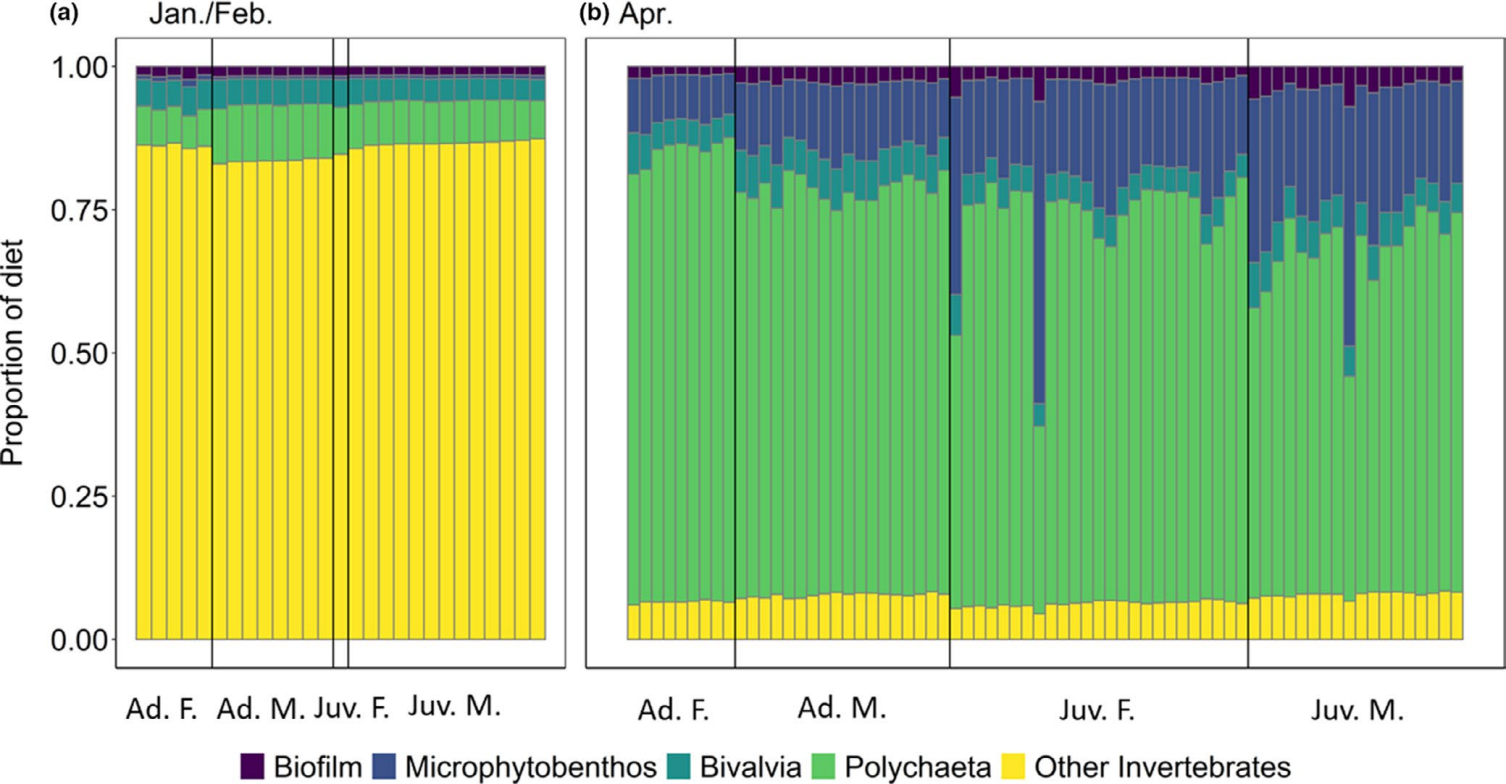
The proportional contributions estimated with stable isotope mixing models of five prey groups (biofilm, microphytobenthos, Bivalvia, Polychaeta, and other invertebrates) to the diets of western sandpipers (*Calidris mauri*) from four demographic groups (adult female = Ad. F., adult male = Ad. M., juvenile female = Juv. F., and juvenile male = Juv. M.) captured in (a) January/February and (b) April in San Francisco Bay, CA, USA. Discrimination factors for dunlin (*C. alpina*) plasma from Evans Ogden et al. (*Δ* ^13^C ± standard deviation (*SD*) = 0.50 ± 0.42‰, *Δ* ^15^N ± *SD* = 3.30 ± 0.32‰; 2004) were used to adjust prey isotope values. See Table S1 for sample sizes

Discrimination factors from Evans Ogden et al. (2004) and Lourenço et al. (2015) were similar, yielding only minor differences in mixing model results, especially in spring when the average percent contribution of each prey group differed between models by less than 2% (Figure 2; Figure S2). In winter, results from the mixing model that used the discrimination factor of Evans Ogden et al. (2004) indicated that western sandpipers consumed an average of 8% Polychaeta and 85% other invertebrates compared to 4% and 91%, respectively, from the mixing model that used the discrimination factor of Lourenço et al. (2015; Figure 2; Figure S2). The average percent contribution differed by less than 1% for the remaining prey groups. (Figure 2; Figure S2). We present the results of subsequent analyses using the mixing model with the discrimination factors of Evans Ogden et al. (2004); results using the mixing model with the discrimination factors of Lourenço et al. (2015) are presented in the supporting information.

**FIGURE 3:**
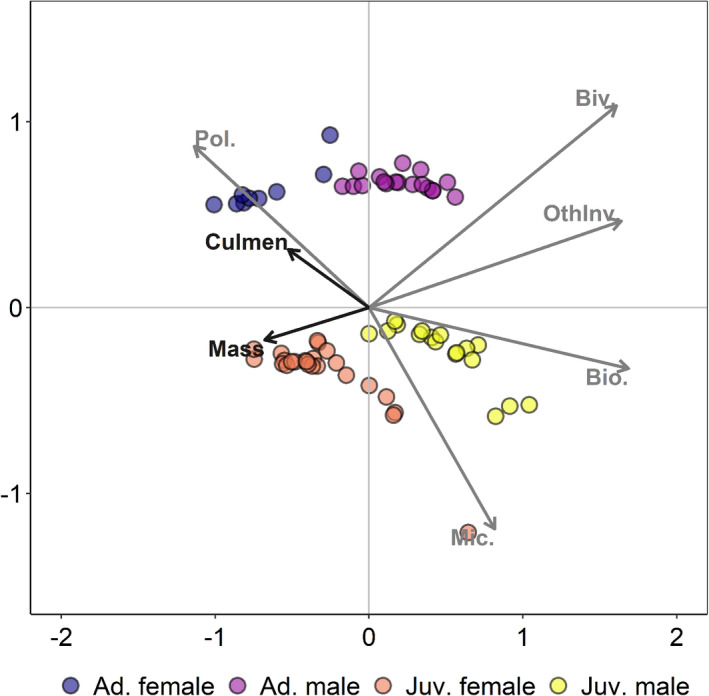
Redundancy analysis (RDA) biplot of the relationships between diet composition and morphometrics in western sandpipers (*Calidris mauri*) from San Francisco Bay, CA, USA in April at the onset of spring migration. Bill (culmen) length (mm) and body mass (g) of sandpipers explained 31% of the variation in diet composition. The proportional contributions of five prey groups (biofilm, microphytobenthos, Bivalvia, Polychaeta, and other invertebrates) to sandpiper diets were estimated using a stable isotope mixing model. Isotope values of prey were adjusted using the discrimination factors of Evans Ogden et al. (*Δ* ^13^C ± standard deviation (*SD*) = 0.50 ± 0.42‰, *Δ* ^15^N ± *SD* = 3.30 ± 0.32‰; 2004). See Table S1 for sample sizes

The diet composition of western sandpipers estimated by the mixing model differed significantly between seasons (*pseudo‐F* = 3,040.86, *R* ^2^ = .95, *p* < .001), among demographic groups (*pseudo‐F* = 37.04, *R* ^2^ = .01, *p* < .001), and among demographic groups within each season (*pseudo‐F* = 26.15, *R* ^2^ = .01, *p* < .001; Table S2). The difference between seasons explained 95% of the variation in western sandpiper diets, whereas the differences among demographic groups and among demographic groups within each season each explained 1% of the variation in diet. The proportions of biofilm and Bivalvia in western sandpiper diets were similar between seasons (Figure 2). Western sandpipers from all demographic groups consumed a large proportion of other invertebrates in winter, whereas they consumed greater proportions of Polychaeta and microphytobenthos in spring (Figure 2). However, the shift from diets dominated by other invertebrates in winter to diets dominated by Polychaeta in spring observed in all demographic groups should be interpreted with caution; this shift could be an artifact of the substantial overlap in stable isotope values between Polychaeta and other invertebrates in spring (Figure 1). In contrast, the stable isotope values of microphytobenthos had minimal overlap with other prey groups during both seasons (Figure 1). In winter, the average consumption of biofilm and microphytobenthos combined was approximately 2% of the diet of western sandpipers regardless of demographic group, whereas in spring, biofilm and microphytobenthos comprised 9%, 13%, 19%, and 24% of the diets of adult females, adult males, juvenile females, and juvenile males, respectively. Some juveniles consumed a large proportion of microphytobenthos in spring (Figures 2 and 3). The mixing model indicated that the combined contribution of biofilm and microphytobenthos made up greater than 25% of the diet in 13 of 70 (19%) western sandpipers captured in spring; of these 13 individuals, all were juveniles, and 9 (69%) were juvenile males (Figure 2).

In winter, there was little variation in prey consumption among demographic groups (Figure 2); therefore, we did not analyze these data with an RDA. In contrast, diet composition differed among demographic groups in spring (Figure 3; Figure S3). Bill length and body mass were both selected as significant variables that explained differences in spring diet composition among western sandpipers (Figure 3). Together, these variables explained 31% of the total variation (*R* ^2^_ad_ *_j_* = 0.31). Bill length and body mass were significantly greater in female western sandpipers compared to males (all *p* < .05), but, within each sex, morphometric measurements did not differ significantly between age classes (all *p* > .05). Consumption of Polychaeta increased with increasing bill length and was greatest in adult females (Figure 3). In contrast, consumption of microphytobenthos increased with decreasing bill length and was greater in juveniles, particularly juvenile males (Figure 3). In addition, males with lesser body mass consumed a greater proportion of other invertebrates compared to females (Figure 3).

## 4 DISCUSSION

Our results provide the first evidence of seasonal differences in diet composition among demographic groups of western sandpipers, supporting the conclusion that age‐ and sex‐related dietary specialization facilitate seasonal resource partitioning in this species. In spring, juveniles, particularly juvenile males, consumed more biofilm and microphytobenthos than adults, supporting our hypothesis that sandpipers with shorter bills, lesser mass, and less prey handling experience would consume the greatest amount of biofilm and microphytobenthos. Further, differences in diet composition among demographic groups were more pronounced at the onset of migration in spring compared to mid‐winter following the expectation that the consumption of biofilm and microphytobenthos provides energy for sandpipers during their breeding migration and may reduce competition when high densities of birds occupy foraging sites.

### 4.1 Age‐related dietary specialization

Differences in morphology, social status, and acquired skills among shorebirds of different age classes can facilitate resource partitioning (Alves et al., 2013; Catry et al., 2012; Recher, 1966; Stein et al., 2008). Although morphological differences between juveniles and adults may influence prey selection in some shorebird species, we did not observe significant differences in bill length or body mass between juvenile and adult western sandpipers within each sex (Durell, 2000). Therefore, a difference in morphology between age classes is an unlikely explanation for the differences that we observed in diet composition between juveniles and adults. However, differences in social status between juvenile and adult western sandpipers could lead to differences in diet composition between age classes. Direct evidence of adult dominance in western sandpipers is lacking, but social status could play a role in the differential use of winter foraging habitats between juveniles and adults (Buenrostra et al., 1999; Fernández & Lank, 2006; Nebel et al., 2002; Warnock & Takekawa, 1995). In SF Bay during spring, juveniles may focus their foraging efforts on biofilm and microphytobenthos because they are excluded by adults from sites with more profitable invertebrate prey. Age‐related differences in acquired foraging skills may also play a role in prey selection in western sandpipers. Juvenile birds tend to forage less efficiently than adults, and evidence from several species of shorebirds, including Eurasian oystercatchers (*Haematopus ostralegus*), ruddy turnstones (*Arenaria interpres*), and black‐necked stilts (*Himantopus mexicanus*), suggests that foraging ability improves with age (Burger, 1980; Durell, 2000; Goss‐Custard & Durell, 1987; Groves, 1978). If juvenile western sandpipers are less adept foragers than adults, juveniles may consume more biofilm and microphytobenthos in spring as a bet‐hedging strategy; biofilm and microphytobenthos offer a readily available and highly abundant energy source that could be used by juveniles to secure adequate nutrition to prepare for migration (Schnurr et al., 2019; Stal, 2003; Underwood & Paterson, 2003).

### 4.2 Sex‐related dietary specialization

While many previous studies have observed differences in foraging behavior and invertebrate prey selection between sexes in western sandpipers, we report the first observation of differences in biofilm and microphytobenthos consumption between sexes (Fernández & Lank, 2008; Mathot & Elner, 2004; Mathot et al., 2010; Nebel, 2005). Our results indicated that consumption of microphytobenthos increased and consumption of Polychaeta decreased with decreasing bill length in western sandpipers. We also found that the consumption of other invertebrates—a prey group predominantly composed of epifaunal invertebrates in the classes Malacostraca and Gastropoda—increased with decreasing body mass. Male western sandpipers have shorter bills and lesser mass than their female counterparts which may make males better adapted, both mechanically and energetically, to consume biofilm, microphytobenthos, and invertebrates on the sediment surface (Elner et al., 2005; Nebel et al., 2005; Sutherland et al., 2000). In contrast, females, with their longer bills, may be better adapted to probe for submerged invertebrates, including Polychaeta (Nebel et al., 2005; Sutherland et al., 2000). Smaller‐bodied males could also be excluded from foraging on more profitable prey, such as Polychaeta, by larger‐bodied females, as has been observed in bar‐tailed godwits (*Limosa lapponica*; Alves et al., 2013; Both et al., 2003). These results are consistent with previous studies (Alves et al., 2013; Both et al., 2003; Elner et al., 2005; Nebel et al., 2005; Sutherland et al., 2000); however, the dietary contributions in our study were inferred from stable isotope values. This indirect measure of diet composition is less accurate when stable isotope values of prey groups overlap, which was the case for Polychaeta and other invertebrates in spring. Future studies that employ direct measures of diet composition, such as stomach content or fecal DNA analysis, would help confirm the differences in diet composition that we observed between seasons and among demographic groups.

### 4.3 Seasonal resource partitioning

Coincident with previous research, we did not detect differences in diet composition among demographic groups of western sandpipers during mid‐winter, though inferences about the winter diet of juvenile females were limited by a small sample size (Franks et al., 2013). In spring, Beninger et al. (2011) found that western sandpipers shifted their diets toward biofilm and microphytobenthos. We also observed an increase in biofilm and microphytobenthos consumption in spring. Further, our results indicated that the magnitude of this shift differed among demographic groups. This seasonal change in diet composition could have been driven by several factors. Seasonal shifts in prey abundance resulting from changing environmental conditions and prey population dynamics could influence diet composition (Rowan, 2012). For example, in SF Bay polychaetes (>20 mm) and small bivalves (≤6 mm) within the size classes consumed by western sandpipers are present in greater densities in January than in April (Rowan, 2012). In contrast, biofilm biomass is greater in spring than in winter due to factors such as a longer photoperiod, greater photon flux density, and greater temperature (Guarini et al., 1998; Schnurr et al., 2019). Accordingly, we observed that western sandpipers from all demographic groups focused their foraging efforts on invertebrates during mid‐winter, whereas in spring, biofilm and microphytobenthos consumption increased in all demographic groups, but especially in juveniles.

Increased inter‐ and intraspecific competition caused by density‐dependent prey depletion could also contribute to seasonal differences in western sandpiper diets. SF Bay is one of the most important foraging areas for migratory shorebirds along the Pacific coast, supporting an average of 52.3% of all migratory shorebirds in the region during spring (Page et al., 1999). High densities of foraging shorebirds could increase interspecific competition for prey in spring. For example, competitive interactions with dunlin at migratory stopover sites are known to influence western sandpiper foraging behavior and may cause western sandpipers to increase their biofilm consumption to reduce competition (Jiménez et al., 2015; Mathot et al., 2010; Senner et al., 1989). Nonavian competitors, including fishes and crabs, could also reduce the available prey biomass in spring (Lovvorn et al., 2013). In addition, intraspecific competition could influence western sandpiper diets. In spring, western sandpipers migrating from southern latitudes begin their migration earlier than those wintering farther north (Bishop et al., 2004; Butler et al., 1987; Nebel et al., 2002). This pattern of differential migration leads to an increased abundance of western sandpipers in SF Bay in April relative to mid‐winter (Rowan, 2012). At our study site in April, sandpiper abundance can exceed carrying capacity based on invertebrate energy content, and it is likely that other foraging areas in SF Bay become similarly depleted in spring (Rowan, 2012). Our observation that western sandpipers increased their biofilm and microphytobenthos consumption in April could indicate that sandpipers shift their diets in spring in response to density‐dependent increases in inter‐ and intraspecific competition.

As they prepare for their spring migration, western sandpipers undergo changes in physiology and organ morphology that facilitate rapid fattening and help condition birds to endure long‐distance migratory flights (Egeler & Williams, 2000; Guglielmo & Williams, 2003; Stein et al., 2005; Williams et al., 2007). Coincident with the increase in biofilm and microphytobenthos consumption that we observed in all demographic groups in spring, these physiological changes may help western sandpipers to assimilate energy from biofilm and microphytobenthos during migration. Consumption of biofilm and microphytobenthos could provide sandpipers with performance enhancing fatty acids to sustain long‐distance migratory flights. Several studies have documented the importance of n‐3 polyunsaturated fatty acids in exercise performance (Maillet & Weber, 2006, 2007). Although biofilm contains low concentrations of these fatty acids, their concentrations peak in spring, and sandpipers could consume a sufficient quantity of biofilm to enhance their migratory performance (Quinn et al., 2017; Schnurr et al., 2019, 2020).

Our inferences about seasonal resource partitioning in western sandpipers rely on the assumption that stable isotope values of western sandpiper plasma were at equilibrium with local SF Bay prey. This assumption is reasonable for all birds captured in winter and most birds captured in spring because western sandpiper plasma should reach isotopic equilibrium in less than 10 days (Lourenço et al., 2015). However, a small number of western sandpipers captured in spring may have been migrants that had recently arrived in SF Bay. The plasma of these migrants would reflect the diet of their previous foraging area south of SF Bay. A range‐wide study of blood stable isotope values indicated that mean *δ* ^15^N values from western sandpipers wintering south of SF Bay ranged from 6.5 to 12.1‰ and were substantially less than the mean *δ* ^15^N values of birds wintering in SF Bay (16.9‰; Franks et al., 2013). Assuming the magnitude of difference is similar in spring, any migrants captured in SF Bay that had not reached isotopic equilibrium would have had *δ* ^15^N values that were much less enriched than other western sandpipers in our sample. It is likely that these migrants would have been identified as outliers; however, if they were included our analysis, our mixing model would have estimated a high percent contribution of biofilm and microphytobenthos to the diet of these birds because of their less enriched *δ* ^15^N values.

### 4.4 Implications of resource partitioning among demographic groups

Resource partitioning among demographic groups has important implications for the ecology, evolution, and conservation of a species. Dietary specialization can influence a suite of ecological interactions including the intensity of inter‐ and intraspecific competition, microhabitat use, and the risk of predation or parasitism. In particular, different mortality risks among demographic groups specializing on different prey taxa could markedly impact the size and demographic structure of a population (Durell, 2000, 2003). For example, certain prey may supply greater nutrition, while others have a lower risk of predation or parasitism. This risk‐benefit trade‐off could lead to differences in body condition or mortality risk among demographic groups of western sandpipers; however, the implications of dietary specialization are difficult to assess because the physiological processes used to assimilate nutrition from biofilm and differences in predation risk for birds consuming biofilm compared to invertebrates are uncertain. Future studies aimed at understanding the effects of biofilm consumption on body condition and the risk‐benefit trade‐offs of different diets are warranted.

From an evolutionary perspective, differences in body condition or mortality risk resulting from dietary specialization can impact an individual's fitness (Bolnick et al., 2003; Durell, 2000). In addition, intraspecific niche variation could subject demographic groups to different selection pressures and facilitate adaptive speciation (Bolnick et al., 2003). However, sympatric speciation appears unlikely because the differences in diet composition that we observed among demographic groups of western sandpipers were subtle and seasonal, and theoretical models of competitive speciation have demonstrated that niche variation between individuals needs to be relatively large to drive speciation (Dieckmann & Doebeli, 1999).

Understanding the ecological and evolutionary consequences of resource partitioning among demographic groups can help resource managers make informed conservation decisions. If a demographic group specializes in foraging within a particular habitat or on particular prey, the loss of that habitat or prey could have a disproportionately negative affect on the demographic group. Although the importance of conserving invertebrate prey for migratory shorebirds has been well‐recognized, conservation strategies that target regions of high biofilm production are lacking. For western sandpipers, conservation of biofilm at migratory stopover sites as far south as SF Bay could help support the nutritional demands of all demographic groups.

## 5 CONCLUSION

During the nonbreeding season, shorebirds rely on adequate prey at wintering and migratory stopover sites to maintain their body condition and prepare for breeding. With high metabolic rates and long migration distances, they must forage on nutrient‐dense prey to meet their daily energy requirements. Increased competition for prey during migration, a period of extremely high energy expenditure, could result in age‐ and sex‐related dietary specialization. Our results provide the first evidence that age‐ and sex‐related dietary specialization in western sandpipers facilitate seasonal resource partitioning that could reduce competition during spring at the onset of the breeding migration. Seasonal dietary specialization in western sandpipers was associated with differences in bill length and body mass. Differences in social status and acquired foraging skills may also play a role. The factors driving dietary specialization are likely to vary temporally for many species. Our study underscores the importance of examining resource partitioning throughout the annual cycle to inform fitness and demographic models and facilitate conservation efforts.

## CONFLICT OF INTEREST

The authors declare no conflicts of interest.

## AUTHOR CONTRIBUTION

**Laurie A. Hall:** Formal analysis (lead); Writing‐original draft (lead); Writing‐review & editing (lead). **Susan E. W. De La Cruz:** Conceptualization (equal); Formal analysis (supporting); Funding acquisition (equal); Investigation (equal); Methodology (supporting); Project administration (equal); Resources (lead); Supervision (equal); Writing‐original draft (supporting); Writing‐review & editing (supporting). **Isa Woo:** Conceptualization (equal); Data curation (lead); Formal analysis (supporting); Investigation (equal); Methodology (supporting); Project administration (lead); Writing‐original draft (supporting); Writing‐review & editing (supporting). **Tomohiro Kuwae:** Data curation (supporting); Funding acquisition (equal); Methodology (supporting); Writing‐original draft (supporting); Writing‐review & editing (supporting). **John Y. Takekawa:** Conceptualization (lead); Funding acquisition (lead); Investigation (lead); Methodology (lead); Project administration (equal); Supervision (equal); Writing‐original draft (supporting); Writing‐review & editing (supporting).

## Supporting information

Appendix S1

## Data Availability

Data from this research are archived at Hall et al. (2020). Western sandpiper diet composition in south San Francisco Bay, CA. U.S. Geological Survey Data Release. https://doi.org/10.5066/P9XWNJRI.
