# Supplementary material for: Age‐ and sex‐related dietary specialization facilitate seasonal resource partitioning in a migratory shorebird
Source: Ecol Evol. 2021 Jan 20;11(4):1866–76. doi: 10.1002/ece3.7175 (PMC7882968; doi:10.1002/ece3.7175)
Supplement: Supplementary file 1 — Appendix S1 [file ECE3-11-1866-s001.docx]

SUPPORTING INFORMATION

Age- and sex-related dietary specialization facilitate seasonal resource partitioning in a migratory shorebird

Laurie A. Hall^1^, Susan E. W. De La Cruz^1^, Isa Woo^1^, Tomohiro Kuwae^2^, John Y. Takekawa^1,3^

Results of analyses examining differences in diet composition among demographic groups of western sandpipers (*Calidris mauri*) from San Francisco Bay, CA. USA.

Table S1. Sample sizes for western sandpipers (*Calidris mauri*) and five prey groups. Samples were collected in January/February and April in San Francisco Bay, CA, USA.

|  | Jan/Feb | Apr |
| --- | --- | --- |
| Western sandpiper | *n* | *n* |
| Adult female | 5 | 9 |
| Adult male | 8 | 18 |
| Juvenile female | 1 | 25 |
| Juvenile male | 13 | 18 |
| Prey |  |  |
| Biofilm | 9 | 9 |
| Microphytobenthos | 9 | 9 |
| Bivalvia | 16 | 15 |
| Other invertebrates | 12 | 18 |
| Polychaeta | 9 | 13 |

Table S2. Results of a permutational multivariate analysis of variance (PERMANOVA) estimating the effects of season (mid-winter or spring), demographic group (adult female, adult male, juvenile female, juvenile male), and their interaction on diet composition of western sandpipers (*Calidris mauri*) in San Francisco Bay, CA, USA. Results for each effect include: the degrees of freedom (*df*), the sums of squares (*SS*), the mean square error (*MS*), the pseudo-F statistic (*F*), the proportion of explained variance (*R^2^*), and the p-value (*p*). The proportional contributions of five prey groups (biofilm, microphytobenthos, Bivalvia, Polychaeta, and other invertebrates) to the diets of individual sandpipers were estimated with a stable isotope mixing model using *δ*^13^C and *δ*^15^N values of sandpiper plasma and prey. Isotope values of prey were adjusted using the discrimination factors of Lourenço et al. (*Δ*^13^C ± SD = 0.32 ± 0.16 *‰*, *Δ*^15^N ± SD = 3.30 ± 0.20 *‰*; 2015).

| Effect | *df* | *SS* | *MS* | *F* | *R^2^* | *p* |
| --- | --- | --- | --- | --- | --- | --- |
| Season | 1 | 13.52 | 13.52 | 3112.98 | 0.95 | 0.001 |
| Demo. group | 1 | 0.16 | 0.16 | 35.75 | 0.01 | 0.001 |
| Season x Demo. group | 1 | 0.10 | 0.10 | 22.97 | 0.01 | 0.001 |
| Residuals | 93 | 0.40 | 0.00 | NA | 0.03 | NA |
| Total | 96 | 14.18 | NA | NA | 1.00 | NA |


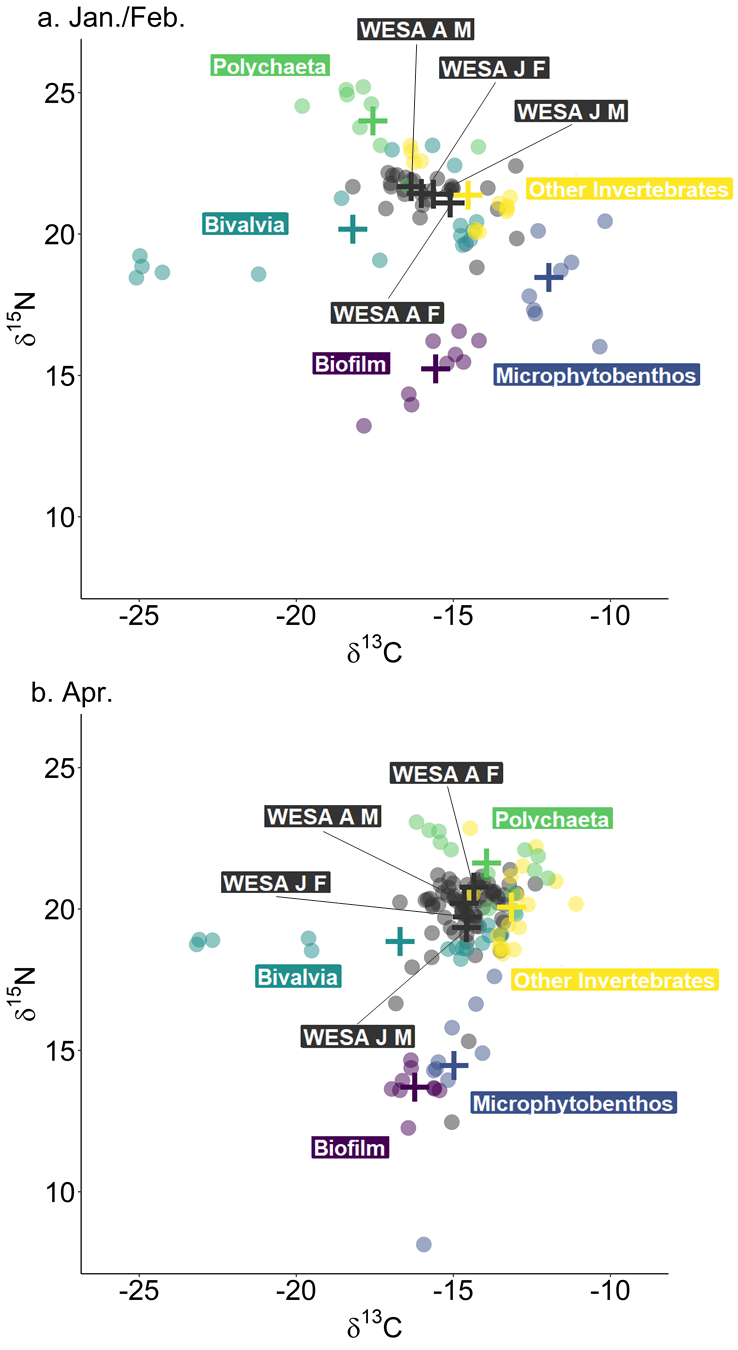


Fig. S1. Stable *δ*^13^C and *δ*^15^N isotope values of western sandpiper (*Calidris mauri*; WESA A F = adult female, WESA A M = adult male, WESA J F = juvenile female, WESA J M = juvenile male) plasma and five prey groups collected in (a.) January and February and (b.) April in San Francisco Bay, CA, USA. Prey isotope values were adjusted by adding discrimination factors for dunlin (*C. alpina*) plasma from Lourenço et al. (*Δ*^13^C ± SD = 0.32 ± 0.16 *‰*, *Δ*^15^N ± SD = 3.30 ± 0.20 *‰*; 2015). Means for each group are displayed as crosses. See Appendix Table A1 for sample sizes.


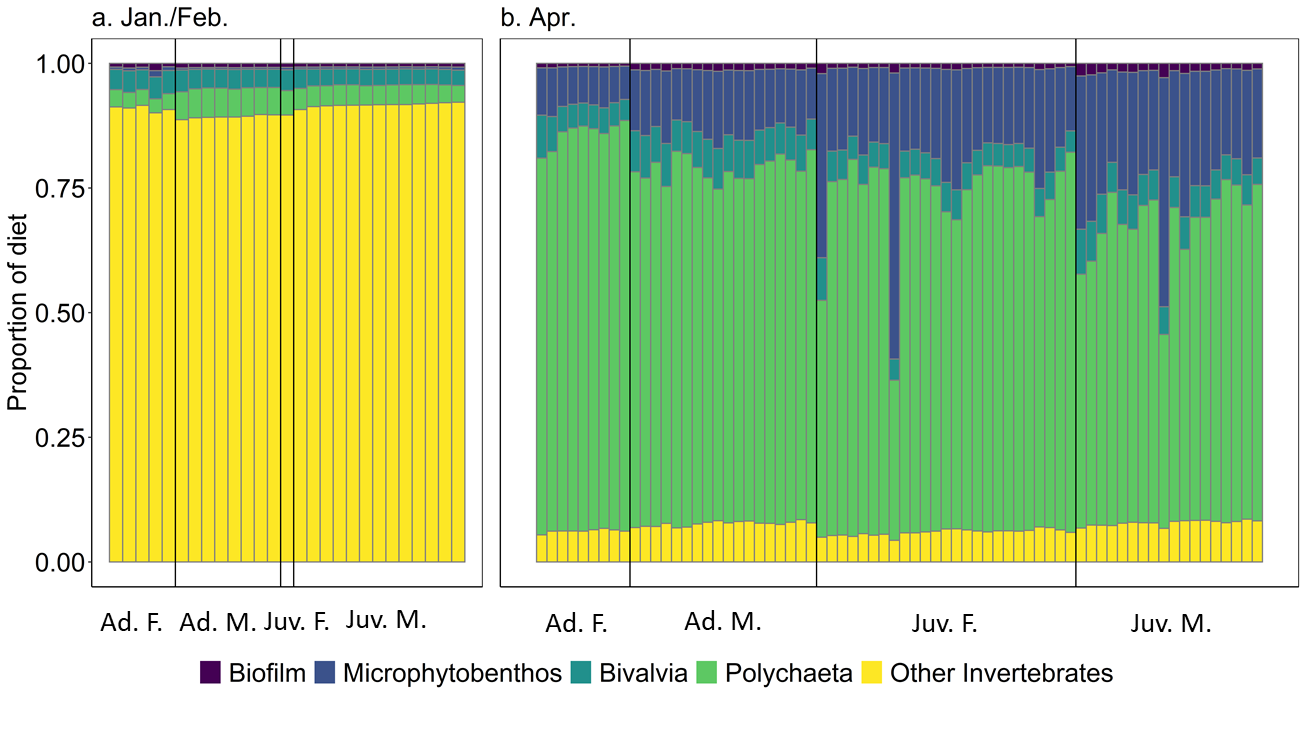


Figure S2. The proportional contributions estimated with stable isotope mixing models of five prey groups (biofilm, microphytobenthos, Bivalvia, Polychaeta, and other invertebrates) to the diets of western sandpipers (*Calidris mauri*) from four demographic groups (adult female = Ad. F., adult male = Ad. M., juvenile female = Juv. F., and juvenile male = Juv. M.) captured in (a.) January/February and (b.) April in San Francisco Bay, CA, USA. Discrimination factors for dunlin (*C. alpina*) plasma from Lourenço et al. (*Δ*^13^C ± SD = 0.32 ± 0.16 *‰*, *Δ*^15^N ± SD = 3.30 ± 0.20 *‰*; 2015) were used to adjust prey isotope values. See Appendix Table A1 for sample sizes.


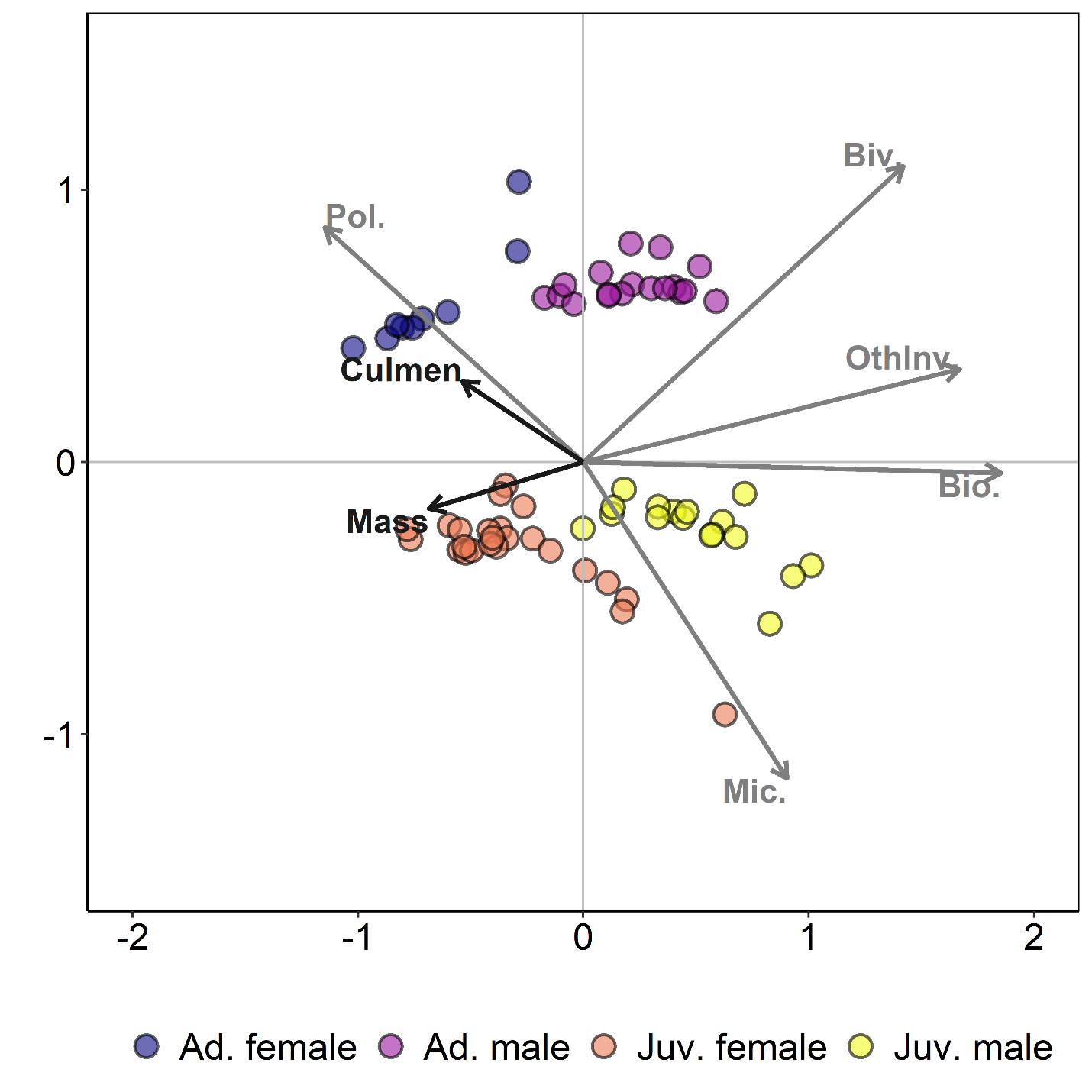


Fig. S3. Redundancy analysis (RDA) biplot of the relationships between diet composition and morphometrics in western sandpipers (*Calidris mauri*) from San Francisco Bay, CA, USA in April at the onset of spring migration. Bill (culmen) length (mm) and body mass (g) of sandpipers explained 33% of the variation in diet composition. The proportional contributions of five prey groups (biofilm, microphytobenthos, Bivalvia, Polychaeta, and other invertebrates) to sandpiper diets were estimated using a stable isotope mixing model. Isotope values of prey were adjusted using the discrimination factors of Lourenço et al. (*Δ*^13^C ± SD = 0.32 ± 0.16 *‰*, *Δ*^15^N ± SD = 3.30 ± 0.20 *‰*; 2015). See Appendix Table A1 for sample sizes.

References

Lourenço, P. M., Granadeiro, J. P., Guilherme, J. L., & Catry, T. (2015). Turnover rates of stable isotopes in avian blood and toenails: Implications for dietary and migration studies. *Journal of Experimental Marine Biology and Ecology*, *472*, 89–96. doi: 10.1016/j.jembe.2015.07.006
